# The Importance of Biobehavioral Research to Examine the Physiological Effects of Racial and Ethnic Discrimination in the Latinx Population

**DOI:** 10.3389/fpubh.2021.762735

**Published:** 2022-01-10

**Authors:** Airín D. Martínez, Evelyn Mercado, Marielena Barbieri, Su Yeong Kim, Douglas A. Granger

**Affiliations:** ^1^Department of Health Promotion and Policy, School of Public Health and Health Sciences, University of Massachusetts-Amherst, Amherst, MA, United States; ^2^Department of Psychological and Brain Sciences, College of Natural Sciences, University of Massachusetts-Amherst, Amherst, MA, United States; ^3^Department of Human Development and Family Sciences, University of Texas-Austin, Austin, TX, United States; ^4^Institute for Interdisciplinary Salivary Bioscience Research, University of California, Irvine, Irvine, CA, United States; ^5^The Johns Hopkins University Bloomberg School of Public Health, School of Nursing, School of Medicine, Baltimore, MD, United States; ^6^Saliva Bioscience Laboratory, University of Nebraska-Lincoln, Lincoln, NE, United States

**Keywords:** biobehavioral research, ethnic discrimination, immigrants, Hispanic, Latinos, Latinx, racial discrimination, racism

## Abstract

A growing body of research is documenting how racial and ethnic populations embody social inequalities throughout the life course. Some scholars recommend the integration of biospecimens representing the hypothalamic-pituitary-adrenal axis, neurological and endocrinological processes, and inflammation to capture the embodiment of inequality. However, in comparison to other racial and ethnic groups, there has been little research examining how Hispanic/Latinx persons embody racial and ethnic discrimination, much less resulting from institutional and structural racism. We provide a rationale for expanding biobehavioral research examining the physiological consequences of racism among Latinx persons. We identify gaps and make recommendations for a future research agenda in which biobehavioral research can expand knowledge about chronic disease inequities among Latinx populations and inform behavioral and institutional interventions. We end by cautioning readers to approach the recommendations in this article as a call to expand the embodiment of racism research to include the diverse Latinx population as the United States addresses racial inequity.

## Introduction

Racism is an ideology that asserts a group as inherently superior to others and that perpetuates a system of domination (1). There are four levels of racism: internalized, interpersonal, institutional and structural racism. Racism in the Americas is grounded in racial capitalism, in which capital accumulation exists through racial exploitation, and white supremacy, a system and paradigm that produces Eurocentrism and white skin as superior to all other paradigms and darker skin (2). Racism is manifested through the historic unequal access to economic opportunities, health care, political participation, and safety for racial and ethnic minorities. Ethnic and racial discrimination refers to the systematic unfair treatment of non-white persons based on phenotype (skin color, hair texture, accent, and other physical features) and/or self-identified ethnic and racial background (3).

There have been requests to examine how racial and ethnic minorities embody discrimination and systemic inequalities throughout the life course (4–7). Biospecimens are often used in biobehavioral research to demonstrate how diverse acute and chronic stressors from institutionalized and interpersonal racism are related to physiological responses to stress and the etiology of chronic disease specifically, and racial and ethnic health disparities generally (8, 9). However, in comparison to other racial and ethnic groups in the United States (U.S.), there has been little biobehavioral research examining how Latinx persons embody racial discrimination, with few exceptions [e.g., (10–14)].

We emphasize institutionalized racism, or racism that results from often colorblind policies and historic, institutionalized practices that may not specifically target racial or ethnic minorities but produce negative consequences on marginalized racial and ethnic populations' economic and political advancement. Since the founding of the United States, there has been institutional racism that has excluded, and continues to exclude, Latinx and Mexican-origin persons (particularly, U.S. citizen Latinx persons) from being integrated into U.S. society (15) through civic engagement, political participation, economic mobility, and intense policing targeting Latinx persons. Institutional racism from diverse institutions together produces relational and supportive forms of structural racism (16). Structural racism is a system of policies, institutional practices, and cultural representations that develop and maintain racial hierarchies; specifically, in which Whites are the advantageous group in U.S. society. Structural racism promotes adverse health effects beyond the interpersonal level by reducing racial-ethnic minorities' economic opportunities and by producing social isolation from the mainstream society (17).

The main goal of this paper is to provide the rationale for expanding biobehavioral research examining the physiological consequences of racial and ethnic discrimination among Latinx persons. This paper comes at a time when Latinx persons in the United States are experiencing the second highest hospitalizations and deaths from COVID-19 (18) and are experiencing high levels of state-sanctioned violence from law enforcement (19) and immigration enforcement operations (20–22). Yet, the current participation of Latinx persons in NIH-funded research is low. Between 2008 and 2015, only 4.4% of all National Institutes of Health research program grants exclusively addressed Latinx health. Cancer and obesity were the most studied health outcomes, and most studies took place in California, Texas, Northeastern U.S. cities, and Illinois (23). As racism is being declared a public health issue by many U.S. states and municipalities, Latinx people need to be part of those conversations and the interventions to reduce the negative effects of racism, given their history with racial discrimination and this group's complex racial and ethnic position in the United States.

Biobehavioral research consists of interdisciplinary research that integrates biological, psychosocial, and behavioral contributors to health to elucidate the mechanisms of disease and symptom etiology. Research created from biobehavioral approaches should inform psychosocial, environmental, and biological interventions that prevent and reverse disease. We identify gaps and make recommendations for a research agenda in which biobehavioral research can expand knowledge about chronic mental and cardiometabolic disease inequities among Latinx populations and inform behavioral and institutional interventions. We demonstrate that biobehavioral research examining the relationship between racial-ethnic discrimination and Latinx health can avoid essentializing Latinx bodies by contextualizing race/ethnicity variables and by disseminating the research findings to critical stakeholders. We end by cautioning readers to approach our recommendations not as an opportunity to exploit Latinx communities for research, but as a call to expand the embodiment of racism research to include the diverse Latinx population as the U.S. begins to address structural racism.

### Hispanic/Latina/o/x in the United States

A discussion about the active use of Hispanic or Latina/o/x is beyond the scope of this article [see (24) for discussion] yet, we will briefly define these terms. *Hispanic* has origins in Spanish colonization to refer to those persons that were born in Spain or were the progeny of Spanish parents. However, in the 1970s *Hispanic* was the label adapted by the Nixon Administration to distinguish those persons that were in the United States, but had descendants from Spanish-speaking, Latin American countries (25) or Spain (26). “Hispanic” was used for the first time in the 1980 U.S. Census (27). Therefore, Hispanic is often interpreted and utilized as an administrative ethnic category imposed on persons with descendants from Spanish-speaking Latin American countries.

“Latin**a/**Latin**o”** has roots in colonial French epistemology, which constructed “Latina/o” to distinguish Anglo-Saxon Americans from non-Anglo-Saxon Americans. Any New World resident that spoke a Latin language (i.e., French, Spanish, or Portuguese) could use the label (26). During the 1970s, Chicana/o/x and Puerto Rican organizers in the 1970s advocated for a categorical designation that distinguished them from the mainstream European Americans (Whites) to account for disparities occurring in their communities. They advocated for “Latina/o” not “Hispanic” (28). Instead, the Office of Management and Budget took King of Spain, Juan Carlos I's recommendation to use “Hispanic” to designate all those persons whose culture or origin is Spanish, regardless of race (29). For the last 36 years, the use of “Latina/o” has become more popular in both everyday vernacular and for legal administrative purposes. The 2000 U.S. Decennial Census aggregated Hispanic with Latino to represent the “Hispanic/Latino” ethnic category widely used today (27). In health research Hispanic and Latina/o are often used interchangeably to refer to persons in the United States who have descendants from Spanish-speaking, Latin American countries or Spain.

Hispanic/Latina/o/x (from now on *Latinx*) are a multiracial, multilingual, and multinational racialized ethnic group, that face ethnic and racial discrimination in the United States and other countries where Latin American and U.S.-born Latinx persons reside (24). There are about 60.6 million Latinx persons in the United States (30) and they have been the largest U.S. racial and ethnic minority group since 2003 (31). In California and Texas, Latinx people make up 45% of the population (30). Four out of five Latinx persons is a U.S. citizen, while the number of immigrants from Latin America has declined since 2007. The majority of Latinx persons in the U.S. are Mexican origin (61.9%), followed by Puerto Ricans (9.7%) and Central Americans (9.5%) (32). Despite the historic presence of Latinx populations in the United States (33), as Zou and Cheryan (34) demonstrate, Latinx populations are perceived as both inferior and as perpetual foreigners by the general public.

It is important to note that racial and ethnic self-identification is personal and complex. The choice to identify with “Hispanic,” “Latina/o,” or “Latinx” varies by an individual's age, country of origin, gender, immigrant generation, sexuality, geographic region of residence [e.g., persons in New Mexico prefer Hispanic (35)], but more importantly, their relationship to white supremacy and U.S. racial codes. For example, Latinx persons that experience internalized racism and reject their African and/or indigenous ancestry, may only identify with their European ancestry. Similarly, some Latinx uncritically take a *mestizaje* (miscegenation) position in which they acknowledge their diverse ancestry (e.g., a descendant of slaves, indigenous persons, and Europeans), but use *mestizaje* to deny their racism and ability to perpetuate white supremacy. Moreover, there can be variations in racial and ethnic self-identification within the same family, in which parents and siblings identify with dissimilar categories to best represent their self-perceptions and their phenotype [e.g., “street race,” (36)] and their political positions. So, the variability in self-identification and intersubjective racial-ethnic narratives problematizes Latinx persons' ability to neatly identify with any of the administrative categories we use in health research. More importantly, the diversity of Latinx persons' racial phenotypes create variability in their exposure to and experiences with structural, institutional and interpersonal racism.

Regardless of the ways that Latinx persons self-identify, they experience ongoing discrimination in education, health care, housing, labor, and financial markets (37), which can be consequential to their health. Using Hispanic Community Health Survey/Study of Latinos data, Arellano-Morales et al. (38) found that lifetime discrimination exposure ranged from 64.9 to 98% across Latinx subgroups. U.S.-born Latinx persons report more racial discrimination than foreign-born Latinx persons, and reports of discrimination varies by institutional context: seeking a variety of health care services (39, 40), seeking employment (41), at work (42), as a student (43), and in the criminal justice system (44, 45), among others.

### Extant Racial Discrimination in Relationship to Hispanic/Latinx Health

Most research examining the relationship between racism and health focuses on interpersonal racism (3, 46–49) or the covert and overt actions toward a person that express prejudice, hate or bias based on race and/or ethnicity (1). Despite the Latinx population being the largest U.S. racial-ethnic minority, research about racial and ethnic discrimination in relationship to Latinx biobehavioral health is a nascent and developing research area (10–14, 50–52). This research demonstrates that more interpersonal racism reported by Latinx persons is related to increased risk for mental health issues, more substance use, and cardiometabolic conditions like obesity, Type II diabetes, and cardiovascular disease (48, 53). Similarly, Latinx youth reporting more racial discrimination report negative mental health outcomes, such as anxiety and depression, and more substance use (47). Racial discrimination and health researchers have largely overlooked Latinx populations because of their administrative designation as an ethnic group (54), the Hispanic/Latino epidemiological paradox (55), and the diverse self-identifications of race and ethnicity among Latinx populations.

### Reasons for the Dearth of Racism and Latinx Health Research

The Hispanic/Latino epidemiological paradox is a concept used to describe an epidemiologic pattern in which Latinx populations have lower socioeconomic status and high rates of uninsured yet have a longer life expectancy (56, 57), better infant health outcomes (58), and some healthier practices than the U.S. non-Hispanic, White population (59). However, the analyses informing the epidemiological paradox generally homogenize all Latinx subgroups and nationalities, in that the samples were primarily capturing Mexican-origin and Mexican migrant persons' data (60). These positive statistics can be attributed to selective migration (61), less acculturation to U.S. society, and for some, genetics (62). The positive life/death statistics (e.g., life expectancy, mortality rates) used to describe the epidemiological paradox overshadow the chronic disease (cardiometabolic, physical and psychiatric) disparities experienced by Latinx persons (63), and the intragroup differences in disease (64, 65) and mortality rates.

Researchers have identified divergences from the Latino epidemiological paradox, with disparities between Latinx subgroups (64) and health disparities between birth cohorts and generational status (66, 67) and demographic characteristics, like skin color and self-identifying as a “Black” Hispanic/Latina/o/x (68, 69). For example, in a systematic review of acculturation and health research on Mexican-origin persons in the United States, Carter-Pokras et al. (70) found that U.S.-born Mexican persons with English language proficiency and higher levels of education had better health outcomes than less educated U.S.-born Mexican persons and Mexican immigrants. Non-white immigrants, including those from Latin America, experience increased health disadvantages the longer they remain in the United States, in which migration health advantages diminish and cumulative exposures to racial-ethnic discrimination increase (71). It is problematic to generalize the health advantages of Latinx immigrants to all Latinx persons if we do not consider other factors and examine chronic disease and mental health outcomes.

Moreover, the Latinx population has been administratively and politically constructed to be the only ethnic group in the U.S. Census (54). Despite the imposition of this ethnic administrative category, persons who self-identify or are perceived as Mexican-origin or Latinx do experience racism and racial discrimination in multiple domains of U.S. society and in their countries of origin, especially if they are Latin Americans that phenotypically present as Black or one of many indigenous groups. Latinx people are equally likely to report experiences of prejudice based on racial inferiority and perceived foreignness (34). Latinx persons experience both racial and ethnic discrimination because both race and ethnicity are often conflated, essentialized categories (72). Yet, we must mention that in many situations White-passing Latinx persons can benefit from white privilege (12, 73).

Racial markers for *Latinidad* that are often studied are skin color (74, 75), physical stature (76), occupational status (77), hair texture (75), and other phenotypical markers and cultural practices (78). For example, Rosas (79) writes that if a White Latinx person speaks English with a Spanish accent, it could be interpreted as a racial marker, that can solicit ethnic-racial discrimination. Racial markers of *Latinidad* are not just self-represented, but also contextual—they depend on how racial markers are read in relation to others' interpretations of whiteness. The further a Latinx person's racial markers are from others' interpretations and representations of whiteness, the more intense their experiences of racial discrimination and social exclusion from rights, recognition and resources.

Research has demonstrated a relationship between Latinx persons' self-reported discrimination and adverse health outcomes [e.g., (10, 80–86)], but few in relationship to biobehavioral health. These measures of discrimination tend to aggregate across all experiences of discrimination without considering specific types of discrimination one may experience (87). For example, contextual factors of discrimination such as sources (e.g., out-group members, in-group members, law enforcement), where these experiences are taking place (e.g., at work, in their community, at the national level through policies), specific forms (e.g., overt vs. subtle experiences), and targets (e.g., personal or vicarious), can vary and impact Latinx health in a variety of ways. Yet, to our knowledge, there is limited research which considers these contextual factors of discrimination on Latinx biobehavioral health and subsequent chronic disease.

Further, the few extant measures of racial discrimination [e.g., Everyday Discrimination Scale (88); Experiences of Discrimination (89); Major Experiences of Discrimination Scale (90); The Racial Microaggressions Scale (91); The Racial and Ethnic Microaggressions Scale (92)] have not been examined extensively with Latinx subgroups in different geographic areas of the United States in a longitudinal manner. Due to this lack of research, the reliability and validity of using these measures within all Latinx subgroups and in certain geographical areas is unknown. This is important because experiences of racial and ethnic discrimination differ by regional histories in the U.S. and whether the municipality or state is a new destination or traditional gateway for Latinx populations (93).

We should expand research on the biobehavioral effects of racism in the Latinx population to: (1) characterize Latinx persons momentary physiological responses to acute (e.g., daily, short-term) experiences of ethnic and racial discrimination, (2) discover pathways in which Latinx populations somaticize chronic stress from ethnic and racial discrimination and cultural, environmental and economic deprivation from structural racism; (3) expand our ability to intervene on the mental health and chronic disease disparities among Latinx persons; and (4) produce evidence that informs equitable social and health policies.

## Rationale for Expanding Biobehavioral Research Examining the Physiological Consequences of Racial and Ethnic Discrimination in Latinx Persons

The Hispanic/Latino epidemiological paradox is often found for health outcomes that are related to life and death (e.g., life expectancy, mortality). However, if we look at cancer outcomes, cardiometabolic disease and disability, Latinx persons suffer from many disparities. For example, preventable cancers are the leading cause of death among Latinx persons in the United States (65, 94). In comparison to other racial and ethnic groups, Latinx persons are also more likely to have higher mortality rates in breast, uterine, cervical, and gastrointestinal cancers (94). The prevalence of obesity is higher for U.S.-born Latinx children (2–11 years old) (95), and Latinx adults than for non-Latinx Black persons (96). Type II diabetes is a serious health issue among Latinx adults, in which it was the third leading cause of death for females and the fourth leading cause of death for males in 2015 (97, 98). Molina and Simón (99) assert that there may be discrepancies in the epidemiological paradox because Latinx subpopulations from lower U.S. socioeconomic statuses may experience discrimination more and have fewer material and social coping strategies to mitigate its health effects. Overemphasizing Latinx subpopulations' healthier epidemiological outcomes may distract researchers and policy makers from structural sources of health inequities such as segregation, immigration enforcement operations, and domestic terrorism that result from blatant xenophobia and racism toward Latinx persons.

Researchers would benefit from understanding how social and physical environments interact with diverse Latinx populations' biobehavioral health to produce this contradictory landscape in which the Latinx population have both impressive health indicators (i.e., life expectancy), but also detrimental chronic disease health disparities. Integrating biobehavioral approaches to the health effects of racism in relation to Latinx health could clarify these contradictions because the onset of chronic diseases results from cumulative exposure to material and political deprivation and chronic stress resulting from institutionalized and interpersonal racial discrimination (100).

### Explore Mechanisms for the Progression of Chronic Disease in Latinx Populations

In this section, we explore the biopsychosocial mechanisms that have been examined in relationship to racism and how biomarkers are integrated in research examining the biobehavioral effects of inequality. Using the example of prediabetes and Type 2 diabetes mellitus (T2DM), which is a serious public health issue in Latinx populations in the United States and Latin America (101, 102), we provide an example of how to examine not only the adverse biobehavioral effects of racism in Latinx populations, but its importance in understanding the progression of chronic disease in this population.

Individual experiences of discrimination can lead to biological dysregulation that impacts disease pathology. Therefore, determining which biological processes (i.e., biomarkers) carry the effects of discrimination “under the skin” is an important step for addressing health inequities in Latinx populations. Technological advances have made the collection of biospecimens less invasive (i.e., saliva samples, hair samples), allowing for the use of biomarkers in research conducted with underrepresented and marginalized communities to grow in the last decade. *Biomarkers* are objective indicators of normal or pathological biological processes (103), and are considered candidates for research exploring mechanisms for the progression of disease in the face of adverse life experiences including institutional and interpersonal racism (48, 49).

**Figure 1:**
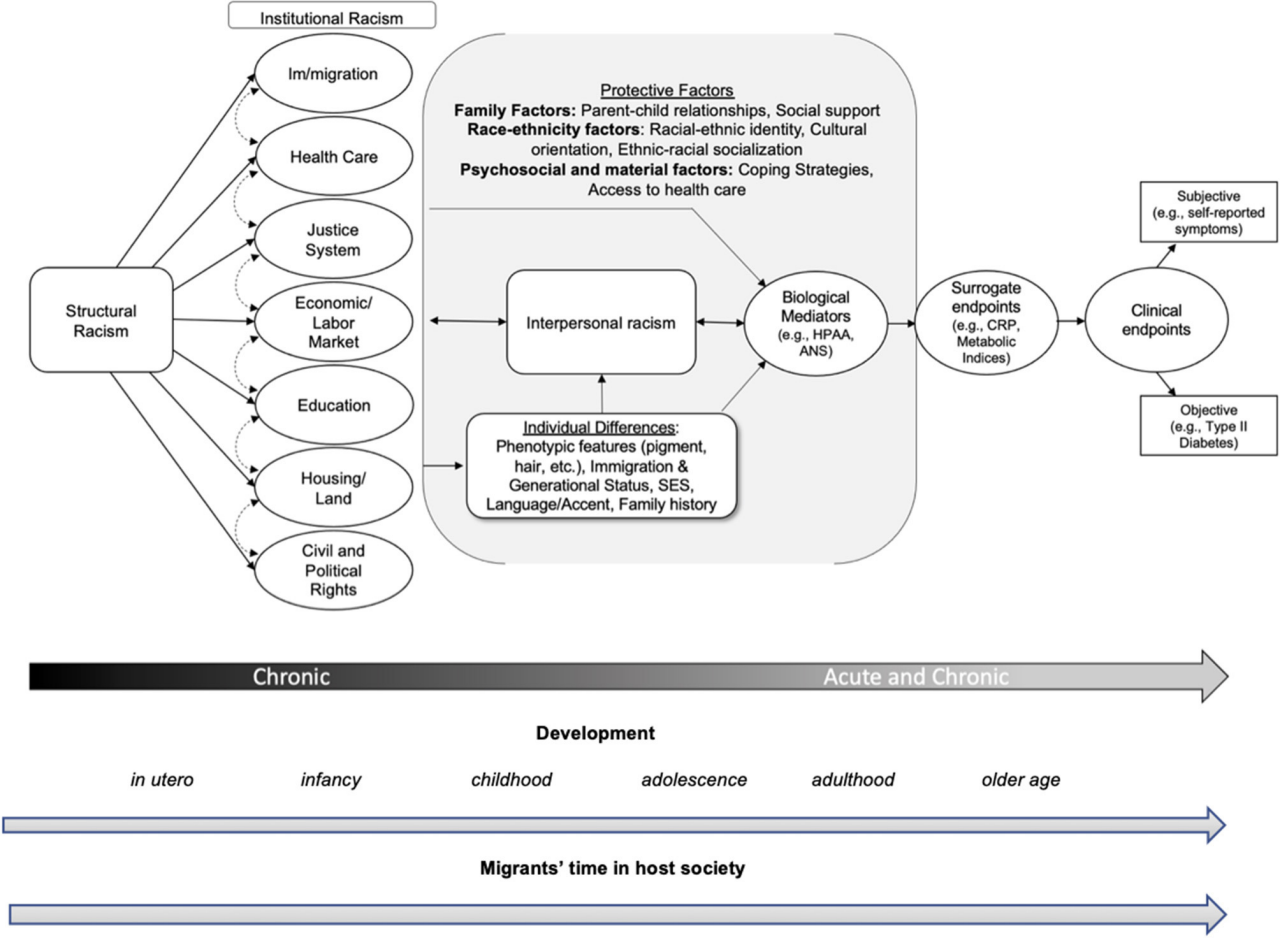
Structural, institutional, and interpersonal racism's effects on biological mediators, surrogate endpoints, and clinical endpoints. This figure demonstrates how structural and institutional racism shape the context in which interpersonal racism occurs. Individual differences are shaped by structural racism, as the meanings for phenotypes in relation to white supremacy, cultural representations of race, racism, and Latinidad are shaped by the structure. Moreover, Latinx life chances, such as their educational attainment and health status, are shaped by the structure. Individual differences in phenotypic features, immigration status, generational status (e.g., first generation), socioeconomic status, language use (indigenous, Spanish, etc.), family history of disease, and accent also shape the ways that structural and institutional racism form experience of interpersonal racism for diverse Latinx persons. Individual differences also influence experience of interpersonal racism from experience. For example, lighter-skinned Latinx person without an accent may not experience interpersonal racism as intensely as a darker-skinned Latinx person. Individual differences, such as family history of disease also play a moderating role on biological mediators, biological surrogates, and clinical endpoints. Dependent on the context and outcomes of interest, these individual differences can directly or indirectly impact the discriminatory experience a Latinx person faces, as well as moderate the link between racism and clinical endpoints. Acute and chronic stress emanating from (a) cultural, environment, and economic deprivation resulting from structural and institutional racism, and (b) experience of interpersonal racism have physiological effects on the body, which manifest as biological mediators [e.g., HPA axis, ANS; see page. 12 of the article and (104)] and surrogate endpoints (e.g., metabolic indices). Over time, as a Latinx child becomes older or an immigrant lives longer in their host society, racial chronic stress can lead to adverse health outcomes and their subsequent clinical endpoints. Protective factors such as familial (e.g., parent-child relationships, social support), race-ethnicity (e.g., identity, cultural orientation, ethnic-racial socialization), and psychosocial and material factors (e.g., coping strategies, health care access), may buffer the impact of structural and institutional racism on biological mediators, which may decrease the likelihood of the development of surrogate endpoints and subsequent subjective (e.g., self-reported depression symptoms) and/or objective (e.g., Type II diabetes) clinical endpoints. The line at the bottom represents time—acute (moments of interpersonal racism) vs. chronic racial stress through ones life course. The institutions capture under structural racism were adapted from Bailey et al. (46) and the biological mediators, surrogate and clinical endpoints were adopted from Robles et al. (104).

Biomarkers can be distinguished into three categories: clinical endpoints, surrogate endpoints, and biological mediators (103, 104). Organizing biomarkers in these three categories allows researchers to effectively and more accurately identify which biological processes health care providers consider to be validated indicators of “health,” “heath status,” and “pathophysiology” (103, 104). *Clinical endpoints* are defined as “a characteristic or variable that reflects how a patient feels, functions, or survives.” These can be objective assessments of disease severity and mortality; an example of a clinical endpoint is the occurrence of a heart attack or hospitalization for a medical condition (103). *Surrogate endpoints* are “a biomarker that is intended to substitute for a clinical endpoint” but may not “assess how a patient feels, functions, or survives” in the same way as a clinical endpoint. To be a surrogate endpoint there must be consistent scientific evidence that the specific biomarker in question (e.g., blood pressure) predicts a clinical outcome [e.g., cardiovascular disease (105)]. Other examples of surrogate endpoints include metabolic syndrome indices (i.e., high fasting plasma glucose, low density cholesterol, elevated waist circumference), that predict increased risk for a host of conditions including T2DM, cardiovascular disease, and stroke [for examples see (104)]. Finally, biomarkers that are not considered clinical or surrogate endpoints are *biological mediators*; biological mediators explain links between psychosocial factors (e.g., discrimination exposure) and underlying biological processes of disease (e.g., surrogate endpoints) but cannot substitute for a clinical endpoint [(106); see below Figure 1].

Some biomarkers can be a clinical endpoint, surrogate endpoint, or biological mediator, depending on the health outcome of interest, for example, uric acid. Hyperuricemia is a clinical endpoint for gout. Elevated levels of serum uric acid in youth are a surrogate endpoint for hypertension (107). Also, uric acid appears to be a biological mediator of hippocampal activity and emotion regulation (108).

Self-reported discrimination exposure has been consistently associated with surrogate endpoints such as BMI, waist circumference, fasting glucose, and blood pressure (38, 109, 110), an association that has also been documented in Latinx immigrants (11, 13, 14). Research on mechanisms linking racial-ethnic discrimination to health have examined biological mediators more extensively compared to biological surrogates or clinical endpoints and have predominantly focused on allostatic biological processes which involve cardiovascular, neuroendocrine, and immune changes that occur in response to stress. Allostasis refers to the altering of baseline biological functioning to facilitate survival in the presence of environmental demands (111); thus, allostatic processes include the biological systems that are activated to respond to stressors (112), including the hypothalamic-pituitary-adrenal axis (HPAA) and autonomic nervous system (ANS). Both the HPAA and ANS have been proposed as major biological pathways linking discrimination-related stress to health outcomes by the Biopsychosocial Model of Racism (8).

The HPAA is a hormone cascade that culminates in the release of the steroid hormone cortisol. HPAA activation is sensitive to stressors characterized as uncontrollable, unpredictable, or socially evaluative, with rises in cortisol levels following exposure to a stressor followed by a decline or recovery, as high cortisol levels lead to activation of receptors in the brain that shut off the response known as a negative feedback loop (113). Importantly, cortisol exhibits a diurnal rhythm, with levels rising sharply in the half hour following awakening (i.e., cortisol awakening response) and gradually declining across the day [i.e., diurnal decline (114, 115)]. It is important to note that chronic experiences of stress have been related to both elevated levels of cortisol (e.g., hyper-arousal) in the body and decreased levels of cortisol (i.e., hypocortisolism), and both cortisol patterns have been associated with disease pathology (116–118). It has been theorized that initial responses to prolonged exposure to a traumatic event or extreme stressor are characterized by sensitization or a “hyper” response. However, prolonged exposure to elevated levels of cortisol may lead to habituation over time and manifest as a blunted or hypo-arousal response (119).

Numerous studies have linked discrimination to alterations in HPAA functioning, both in terms of cortisol reactivity to stress as well as disruptions to diurnal cortisol patterns. Overall, these findings suggest greater exposure to discrimination is linked to both heightened and blunted cortisol reactivity to stress and flatter cortisol diurnal slopes [for review, see (120, 121)]. However, out of those studies, few have included Latinx populations [e.g., (50, 52)], and even fewer have adopted an intersectional approach to examine the physiological impact of discrimination among Latinx people with multiple marginalized identities [e.g., (122)]. For example, in a laboratory study that exposed Latina college students from working-class or middle-class backgrounds to a racially prejudiced interaction partner, only Latinas from middle-class backgrounds exhibited heightened cortisol responses to interacting with a prejudiced partner (122). The authors suggest working-class Latinas may possess more effective coping strategies due to the higher likelihood that they have experienced greater exposure to discrimination compared to middle class Latinas. In a sample of sexually diverse Latinx young adults, exposure to heterosexist and racial discrimination was related to challenges to integrating their sexual and racial-ethnic identity, which in turn, was associated with lower cortisol across the day (123). Taken together these results highlight the importance of accounting for individual differences within the Latinx community.

In contrast to the HPAA, the ANS system is faster acting in the face of stress and less discriminate, regulating energy expenditures and organ functions during and after exposure to a stressor (124). The ANS system includes two branches, the parasympathetic nervous system (PNS) and the sympathetic nervous system (SNS). Sympathetic arousal increases heart rate allowing for mobilization of resources to respond to stress, while parasympathetic arousal modulates heart rate through the vagus nerve, acting as a brake (i.e., vagal brake) to promote active regulation of heart rate (125). Functioning of these branches can be measured through biomarkers such as heart rate variability (HRV; beat-to-beat changes in heart rate caused by PNS and SNS activity), respiratory sinus arrhythmia (RSA; variation in heart rate that occurs during a breathing cycle that is used as indicator of PNS activity), skin conductance levels (SCL; electrodermal activity reflecting SNS activity), cardiac pre-ejection period (PEP; a cardiac contractility index reflecting SNS activity), and circulating levels of catecholamines [i.e., epinephrine and norepinephrine (106, 126)].

ANS functioning has been related to the development of various chronic diseases [e.g., (127, 128)], and is an important biological mediator to consider when documenting the link between discrimination and physical health. Research has highlighted the impact of exposure to discrimination on ANS functioning, for example, exposure to racial discrimination was related to greater PNS activity indexed by high frequency HRV among women who identified as Black or White living with T2DM (129). We strongly recommend measuring both proxies for the HPAA and the ANS when examining racism-related acute and chronic stress.

Additionally, recent research highlights the important role of genetic variation in genes implicated in the biological stress response (e.g., CRHR1, CRHR2, NR3C1), and how epigenetic processes (i.e., modifications to the genome that may lead to changes in gene functionality and phenotypic expression) such as DNA methylation [i.e., the addition of methyl groups to DNA that regulate gene expression; see (130) for discussion on epigenetic research] can impact the functionality of these genes and subsequently stress reactivity [see (131, 132)]. In addition to examining responsivity of the HPAA and ANS, integration of epigenetic processes may yield further clarity on how exposure to racism may contribute to health disparities through changes in HPAA and ANS functionality mediated by differential gene expression. While a growing number of studies have examined epigenetic processes in racial-ethnic minority groups, to our knowledge only one study has examined the link between discrimination exposure (i.e., everyday discrimination exposure) and DNA methylation in a Latinx population (133). The study findings suggest epigenetic markers, namely glucorticoid methylation, is an important risk factor associated with discrimination exposure in Latina mothers born outside of the United States (133).

### An Exemplar of the Biobehavioral Effects of Racism in Latinx Populations

Activation of neuroendocrine (e.g., HPAA) and cardiovascular systems (e.g., ANS) during stressful events have downstream effects on immune functioning (134). While a complete review of the complex nature of communication between the HPA axis and immune systems is beyond the scope of this paper [see (134)], we will provide a very brief example through prediabetes and Type 2 diabetes mellitus (T2DM) of how to examine the biobehavioral effects of structural, institutional and interpersonal racism in relation to T2DM in the Latinx population.

**Figure 2:**
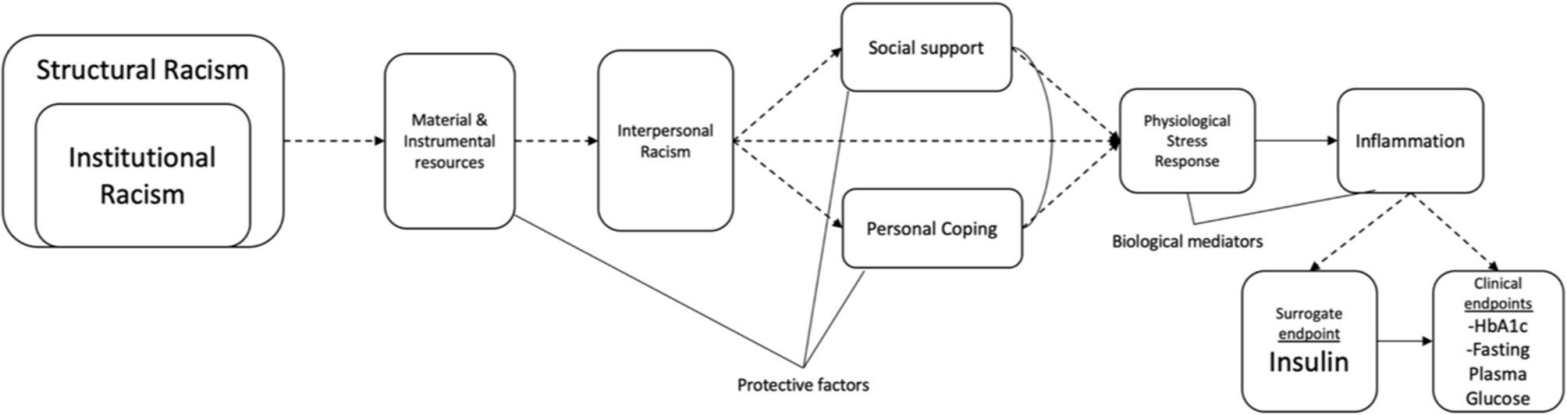
Biobehavioral research examining the racism in relation to clinical surrogates and endpoints of Type 2 diabetes mellitus. This figure presents an example of how to examine the biobehavioral response to racism among Latinx persons and its impact on the development of prediabetes and T2DM. Solid arrows represent established associations and dashed arrows represent potential pathways. Curved lines represent correlations. Solid lines are merely descriptive. For example, insulin has a solid arrow to the clinical endpoints, hemoglobin Alc (HbAlc) and fasting plasma glucose (FPG), since it is established that insulin is the key hormone responsible for bringing glucose into cells and controlling the amount of glucose in the blood. Prediabetes is diagnosed with a HbAlc between 5.7 and 6.4% and a FPG of 100–125 mg/dL. T2DM is diagnosed with a HbA1c >6.5% and a FPG >126 mg/dL. Plasma insulin is usually monitored together with HbA1c and FPG to discern insulin resistance and distinguish between TlDM and T2DM. Both structural and institutional racism shape the economic, educational, legal, environmental, and cultural conditions in which Latinx communities are embedded. In turn, this shapes the food environments, health care access, and living conditions that allow Latinx persons to engage in health behaviors and preventive measures. Nevertheless, material and instrumental resources available to Latinx persons, families, and communities may offset the challenges present in a racist society (16, 46). Structural and institutional racism also produce the conditions and settings in which Latinx people experience interpersonal racism. Social support and personal coping can mediate or moderate the effects of interpersonal racism on physiological stress responses in both the ANS and HPAA stress systems. Here, they are displayed as mediators, assuming that this model is using longitudinal data and the temporality precedence is met (136). If assumptions are not met for a mediation analysis, protective factors against interpersonal racism have been examined as moderators [see (137)]. Acute and chronic racial stress activate immune and inflammatory responses related to β-cell production, pro-inflammatory cytokines (e.g., tumor necrosis factor-α, IL-6, IL-8), and abdominal adiposity. The biopsychosocial pathway was adapted from Hackett and Steptoe (118), Joseph and Golden (138), and Tsenkova et al. (135).

T2DM is a metabolic disease characterized by the inability of the body's cells to receive energy from glucose (i.e., sugar). Insulin is produced by β cells in the pancreas and is the key hormone responsible for bringing glucose into cells and controlling the amount of glucose in the blood. Prediabetes is a condition in which glucose levels are higher than normal, but not high enough to be diagnosed as T2DM. Prediabetes is diagnosed with a hemoglobin A1c (HbA1c; glycated hemoglobin that is chemically linked to sugar) between 5.7 and 6.4% and a fasting plasma glucose (FPG) of 100–125 mg/dL. T2DM is diagnosed with a HbA1c >6.5% and a FPG >126 mg/dL (135). It is estimated that ~88 million people in the U.S. have prediabetes, but most (84%) do not even know they have it (101). Persons who develop T2DM generally have prediabetes and insulin resistance (IR), a situation in which the body's cells do not respond normally to insulin. With the presence of more glucose in the blood, the β cells in the pancreas produce more insulin to attempt to get more glucose into the cells, and over time, the pancreas cannot keep up (101). Plasma insulin is usually monitored together with HbA1c and FPG to discern insulin resistance. Therefore, insulin is a surrogate endpoint for T2DM, while hemoglobin A1c and fasting plasma glucose are clinical endpoints for diagnosing T2DM (see Figure 2).

We chose T2DM because it is a major cause of morbidity, disability, and mortality globally, but especially among Latinx persons in the United States, in which 17% of Latinx persons have T2DM, in comparison to 8% non-Hispanic Whites. Latinx persons in the United States have the highest rates of prediabetes and more than half of the Latinx population is expected to develop T2DM in their lifetime (101). Latinx persons in the United States are 50% more likely to die from T2DM than non-Hispanic Whites (101). Treatment for diabetes is cumbersome with frequent, daily glucose monitoring, and costly due to the expense of these medications, including exogenous insulin (139). In the last 30 years, Latin America and the Caribbean have experienced a steady increase in T2DM prevalence, with the highest prevalence in the Caribbean (17%) and both sides of the U.S.-Mexico border (38.6%). The highest volume of cases in Latin America are in Brazil and Mexico, and most U.S.-based Latinx immigrants are Mexican origin (102). The extent to which racism influences the processes that create prediabetes and the transition to T2DM is worth examining.

Exposure to racial stress activates the HPAA, in which the release of cortisol increases gluconeogenesis and abdominal fat and decreases insulin secretion from the pancreatic β cells (138). Similarly, the frequent release of catecholamines (e.g., norepinephrine and epinephrine) from the ANS system produces insulin resistance, which in turn increases HbA1c, triglycerides, and lowers HDL cholesterol (118, 138). Despite there being few studies linking HPAA or ANS dysregulation to prediabetes and future T2DM, one study using Whitehall II data demonstrated that elevated evening cortisol and flatter cortisol diurnal slopes predicted T2DM 9 years later, independent of behavioral covariates (140).

The release of cortisol during stressful events suppresses the immune system by reducing the expression of pro-inflammatory cytokines [e.g., interleukin (IL)-6] and increasing anti-inflammatory cytokines [e.g., IL-10 (141)]. The development of T2DM from prediabetes could be related to HPAA dysregulation because hypocortisolism is related to the limited release of glucose. Moreover, glucocorticoid receptors on pancreatic β cells may no longer be receptive to cortisol during periods of stress and limited so that no insulin is released, producing hyperglycemia. Acute and chronic stressors produce a physiologic stress response that in turn affects metabolic and inflammatory processes related to the etiology of T2DM (138). Behavioral and biological factors can mediate these biobehavioral responses, while cultural and economic factors could be protective against the biological impact of racism on the body (protective factors discussed below) (see Figure 2).

“Perceived discrimination…has been used to define chronic stress in the diabetes literature, possibly to capture stress-related experiences prevalent among ethnic and racial minorities who are at risk for disparities in diabetes outcomes” (140). Perceived racial discrimination in healthcare and in other settings has been associated with elevated HbA1c levels in adults with T2DM (129, 142, 143). One qualitative study showed that among Latinx immigrants in North Carolina, stress from racial discrimination and documentation status behaviorally complicated participants' ability to regulate their diet (144). Few to no studies have examined how chronic stress from racism is related to the processes that produce prediabetes and its transition to T2DM.

There are a few potential pathways that racism can shape the development of T2DM. One is the material deprivation that results from racialized labor markets and residential segregation, leading to poor access to healthy foods in food-deprived environments produced by structural racism. These conditions can affect the behavioral risk factors for T2DM (i.e., diet and physical activity). Latinx persons living with constant hypervigilance about one's safety from local law enforcement and immigration authorities, also creates chronic stress from institutional racism. The other pathway that racism can shape the etiology of T2DM are the frequency and intensity of experiences with interpersonal racism. The effects of racial stress on T2DM have received less focus partly because T2DM research has focused on behavioral modifications to improve diets and increase activity. Nevertheless, 80% of obese persons do not develop T2DM, and there should be more importance given to negative affect, stress, and psychiatric disorders when examining the progression to T2DM (135).

Further, distinct types of stressors have been found to have different immune effects, with acute stressors enhancing immune functioning while chronic or intense experiences of stress leading to the activation of inflammatory responses as measured by C-reactive protein (CRP), and cytokines such as IL-6 [see (145)]. While inflammation plays an important normative role in the short term by repairing tissue damage, chronic inflammation has been linked to various diseases including cardiometabolic diseases (146). Given the importance of the link between inflammation and stress exposure, research exploring associations between inflammatory markers and exposure to discrimination is imperative for uncovering the etiology of cardiometabolic disease and addressing health inequities [for review see (147)].

In a review of existing literature, both acute and lifetime experiences of discrimination were associated with changes in CRP and IL-6 levels, the most common inflammatory markers measured in discrimination studies (147). All but two of the studies in the review included Latinx population, most focused on African American/Black adults and European American/White adults. Limited studies have examined links between discrimination-related stress and immune functioning among Latinx populations, with few exceptions [e.g., (12)]. We propose conducting studies that monitor Latinx persons with prediabetes and their lifetime and prospective exposure to structural, institutional and interpersonal racism in relationship to their physiological stress responses (in both the HPAA and ANS systems), biological mediators of inflammation (e.g., IL-6, CRP, TNF-α), and their surrogate (insulin) and clinical endpoints (HbA1c, fasting plasma glucose) (see Figure 2). These types of longitudinal, biobehavioral studies would allow us to uncover the progression of chronic diseases and develop interventions to reverse these inequities in Latinx populations.

When allostatic processes are repeatedly activated over time in the face of chronic stress conditions, significant wear and tear develops on the biological systems involved, an outcome coined *allostatic load* (112). Allostatic load (AL) is considered the cumulative effect of stressful life experiences and has documented clinical implications [for review see (148)]. Among Latinx persons, allostatic load has been linked to chronic cardiometabolic conditions. For example, in a study that measured AL in Puerto Ricans living in the United States, higher AL scores were related to obesity, hypertension, diabetes, and cardiovascular disease (149). Interestingly, the link between allostatic load and health conditions in Latinx populations has been found to vary by place of birth, with U.S.-born Latinx persons displaying the highest AL scores followed by foreign-born Latinx persons who have resided in the U.S. longer than 10 years, and the lowest AL scores were documented in foreign-born Latinx persons residing in the United States <10 years (150). Based on a recent review, only 11 studies have examined the effect of discrimination on allostatic load, and of those 11, two included Latinx participants [for review see (151)]. The findings suggest that in 9 out of the 11 studies (152, 153), self-reported discrimination was positively associated with allostatic load (151).

## Discussion

### Future Research in Biobehavioral Effects of Racism in Latinx Populations

While studies have begun to uncover the links between discrimination-related stressors and different types of biomarkers (clinical endpoints, surrogate endpoints, and biological mediators), this work has several limitations that inform future directions. First, most work examines a single biomarker in isolation despite the reality that these biological systems work together and functioning of one system may be largely dependent on functioning of the other system. This limitation highlights the need to examine coordination across biological systems [e.g., ANS-HPA symmetry (154)] to gain a more comprehensive and accurate understanding of the role of biological mediators in disease progression. We strongly recommend examining more than one biological system in the context of a specific health outcome. For example, previous research suggest symmetry between the SNS and HPAA system are related to generalized anxiety disorders [GAD (155)], therefore, to better understand how discrimination may manifest GAD, including both a marker of SNS (e.g., sAA, SCL) and HPA (e.g., cortisol) functioning in study investigations would yield more insight into how these biological mediators function as a pathway linking discrimination to GAD.

Second, researchers need to consider choosing a biomarker that has empirical evidence documenting its association with the health outcome of interest. For example, while cardiovascular (ANS) reactivity is generally associated with cardiovascular disease, evidence of this link varies based on the marker of ANS functioning/cardiovascular reactivity included in each study; blood pressure reactivity is more consistently linked to cardiovascular risk while changes in heart rate variability have been less consistently associated with cardiovascular risk (156). As researchers decide to include biomarkers in research on Latinx populations it is important to ensure that the biomarker can be measured through the collection of a specimen (e.g., saliva) deemed culturally acceptable in the community (157). Latinx persons may be less likely to participate in health-related research if it requires biological specimens (e.g., blood sample, genetic sample) or participants must use invasive medical equipment (158). Likewise, it is very important that the research team identify with the Latinx community and speak the participants' language (English, Spanish, and/or indigenous language) so that they can explain all collection procedures and clarify questions about biospecimen assays. We also suggest giving the participants the option of signing consent forms with pseudonyms or providing verbal consent to further protect their confidentiality, especially if they are afraid of disclosing their documentation status or previous criminal record. Lastly, we recommend using simple visual instructions and reminders about biospecimen collection time, storage, and retrieval, if the participants must collect biospecimens independent of the research team.

Third, research design needs to be seriously considered to address issues with inferring causality. Causality requires a strong theoretical foundation, valid measures, contrast between exposed and unexposed groups, verification, and replication (159, 160). Self-reported measures of interpersonal and institutional racial discrimination regressed onto individual biomarkers alone cannot allow us to infer causality. In this paper, we draw from the biopsychosocial stress model, Latinx ethnic studies, sociological theories, and epidemiological conceptual frameworks to create a set of potential pathways through which structural, institutional and interpersonal racism are related to biological mediators, biological surrogates, and clinical endpoints. Using these theories, hypotheses can be drawn, and we provide a set of covariates (not exhaustive by any means) that should be taken into consideration when examining different levels of racism as it relates to Latinx biobehavioral health (see Figure 1).

To assess differences between Latinx persons exposed to racism vs. those not exposed to racism is complicated. We assume that all Latinx persons are exposed to structural and institutional racism in the U.S., even if they do not acknowledge experiences of interpersonal racism. Nonetheless, exposure to interpersonal racism can be captured through experimental designs. For example, Sawyer et al. (161) employed an experimental design to examine if Latinx college students show an exacerbated stress response in a stress task if they knew that their White partner held racial prejudices. Latinx were randomly assigned to know whether their White partner was racist or held egalitarian attitudes about racial-ethnic minorities. Latinx who believed that their White partner was racist, reported more stress after the interaction and showed a greater cardiovascular response than did participants who believed that their partner had egalitarian attitudes.

It may be difficult to replicate results to infer causality in multi-sited studies and similar research projects executed in different geographies, if researchers do not take into consideration policy differences between different states [see (159, 160, 162, 163)] and geographic differences in municipalities that are established gateways for Latinx migration, which have enclaves, institutional arrangements and supports for Latinx persons, and those that are new destinations, lack established institutional arrangements and supports for Latinx persons (93).

Extant research has primarily used cross-sectional designs that have captured past experiences of racial discrimination and not experiences of racism in real-time [with an exception, (164)]. These cross-sectional designs focus on documenting the associations between discrimination and biomarkers. However, less research has examined the indirect and direct associations in the same study population. Longitudinal designs can disentangle the direction of effects and capture the full mediating process of discrimination to changes in biological mediators, to surrogate or clinical endpoints (see Figure 1). The measures for discrimination are limited to interpersonal and perceptions of institutional racism (3).

Self-reported measures of racism may be subjective to recall bias due to the retrospective nature of remembering past discrimination experiences (165). To minimize recall errors, future research should consider incorporating shorter recollection periods to measure discrimination experiences in questionnaires, use daily diaries to understand the frequency and intensity of racist events (164), as well as conduct interviews with participants prior to administering the survey to help in recollection of past events (165). Alongside these potential methods, biomarkers should be incorporated with these self-reported measures to help ensure internal validation, and confirmatory factor analyses to determine the direct effect of discrimination on Latinx populations health.

Most health research has used the Office of Management and Budget racial and ethnic categories. However, these race and ethnicity variables do not provide a comprehensive assessment of Latinx persons' self-identification, phenotype, accent, and how others perceive their “street race” (36, 166). How others perceive Latinx people's race in critical institutions for livelihood like employment, housing, and health adds covariates that may explain different effects of racism on Latinx biobehavioral health.

Fourth, our final recommendation for future research is to consider sources of resilience and integrate factors that are protective of both the negative effects of racism, at multiple levels, and against the dysregulation of physiological responses to acute and chronic stress originating from racism. While not exhaustive, Figure 1 lists some protective factors that should be considered in future work on Latinx persons. Importantly, the family is a key context to consider while examining biobehavioral processes, and numerous protective factors originate in the family environment (167). The quality of early parenting and family relationships influence the development and responsivity of biological mediators such as the HPA system (168) and the parent-child relationship exerts a stress buffering effect across the lifespan (169). For Latinx persons, prominent cultural values emphasize strong and loyal family ties which result in the use of interdependent coping strategies to reduce the negative effects of racial-ethnic discrimination [e.g., (170)]. Similarly, Kim et al. (51) found that experiencing little discriminatory stress, reporting low parental hostility, and feeling more positive about cultural practices, such as parent language brokering, were protective on cortisol functioning in Latinx adolescents. Three of the most prominent racial-ethnic protective factors against the deleterious effects of racism either originate in the family context and/or interact with family processes: ethnic-racial identity, ethnic racial socialization, and cultural orientation (171, 172).

An important caveat to consider while examining protective factors is the developmental context and timing of the protective effects and their interaction with the biological mediator or endpoint of interest (e.g., Is this factor protective in the short- or long-term? Is there a specific developmental period that this factor is most protective for, like adolescence?) (173). Moving beyond the documentation of the racism-health link to include the biological mechanisms that facilitate that link, as well as protective factors that disrupt the link, are critical directions for future research that aims to develop effective interventions and public policies.

A note of caution, researchers should not document any biobehavioral responses to racial and ethnic discrimination in Latinx populations to map Latinx persons' “inherent biology.” The health outcomes we focus on in this paper are non-communicable chronic diseases, given the disparities in the Latinx population. In large epidemiological studies and systematic reviews, heritable genetic traits generally do account for racial and ethnic disparities in cardiovascular disease (159, 174) or T2DM (175, 176). Three target genome-wide associations (GWAS) have been identified for T2DM, the exemplar chronic disease we apply our conceptual framework to, in select Latinx populations: SLC16A11, HNFA1A, and IGF2 (176). Despite these discoveries, “identified genetic factors explain a small fraction of the estimated heritability” (169, p. 1). For example, socioeconomic factors had a greater impact on explaining the excess prevalence of T2DM among Latinx persons in Boston, not genetic ancestry or self-identified race/ethnicity (175). We do not negate that family history of a specific disease may play a role in developing chronic disease, however, genetic differences often examined in GWAS have selection bias issues related to the reference panels (e.g., they omit Asians represented in the Latinx population), genetic associations could be related to other traits and diseases, and continental (biogeographic) ancestry is not the driver of racism, racial markers of *Latinidad* are i.e., skin color, etc.

The integration of biomarkers documents the short- and long-term physiological consequences of stress from interpersonal, institutional and structural racism that diverse Latinx populations face. We believe there remain many empirical questions related to this, for example, do white-passing Latinx persons experience the same intensity of racial and ethnic discrimination as Black, indigenous or darker Latinx persons? And, if so, in what settings? How does the ethnic-racial discrimination by diverse Latinx persons relate to biobehavioral stress responses? And, how does prolonged experiences with racism manifest as physiological stress? Is the prolonged physiological stress related to behavioral coping strategies and physiological responses that accelerate the chronic disease onset? What protective factors mitigate the effect of racism on physiological functioning, and consequently disease progression?

It is conceptually and methodologically challenging to examine the effects of structural and institutional racism given the lack of longitudinal data, the need to create novel measures to capture diverse racial and ethnic identification and discrimination for Latinx persons, and the immense logistical coordination needed to recruit diverse, Latinx persons at many geographic levels (e.g., census tract, neighborhood, state) and socioeconomic backgrounds. Once the sampling strategy and the measurement of racial and ethnic identification and discrimination are resolved, one way we can analyze the indirect and direct effects of structural racism on clinical endpoints, surrogate endpoints and biological mediators, is to conduct multilevel modeling that a) takes into consideration the relational processes between institutions and b) conducts interactions at multiple levels to capture the direct and indirect effects on individual biobehavioral health.

### The Importance of Examining the Biobehavioral Effects of Racism to Inform Policy

Although many policies such as labor laws, land allocation and political participation, are not directly related to biomarkers, they do shape the environments that Latinx people live in (177), their educational and economic opportunities, and social conditions that they operate in, and subsequently, shape theirs and others' behaviors and emotional responses. We do not simply want researchers to limit their research question to, “Does racism affect Latinx health and in what ways?” We instead want researchers to ask, “In what ways does racism affect diverse Latinx populations in the United States and abroad?” How does the implementation of policies create situations of chronic stress for Latinx populations, and hence, their biobehavioral responses that can lead to chronic disease? Researchers should go beyond identifying the existence of inequities and demonstrate that poor health outcomes are a result of diverse, interlocking policies and institutional practices (i.e., structural racism). Understanding the relationship between policies' consequential environments, economic opportunities, and social conditions and biological mediators, biological surrogates, and clinical endpoints can help us uncover how institutional and structural racism are related to Latinx health inequities. It is at these junctures where we can intervene with community-level interventions or organizational and institutional policies that promote equity.

Intersectionality is an analytic framework that encourages us to examine how different social strata intersect to create interlocking systems of oppression for groups of people in society (178, 179). Intersectionality was coined by Kimberlé Crenshaw to capture the limits of anti-discrimination laws to address black women's discrimination at the workplace across both racial and gender lines. In the vein of Critical Race Theory (CRT), intersectionality assumes that policies produced during the Civil Rights Movements were limited in advancing racial justice, gender equity, and transforming social inequities in U.S. society because colorblind policies and juridical decisions in the United States are key mechanisms of white supremacy. Racism is endemic to U.S. society and operates at all branches of government (36). Intersectionality is of importance when examining the health, and more specifically, the biobehavioral consequences of racism among Latinx persons because it attempts to capture the heterogeneity and multiple marginalized experiences encountered by Latinx persons (54, 180). Moreover, intersectionality like other CRT is about creating policies and laws that both capture (through administrative data collection) and minimize how people's tangible phenotype set them up to have experiences of injustice based on these features (36).

## Conclusion

The goal of this paper was to provide a rationale for expanding biobehavioral research examining the physiological consequences of racial and ethnic discrimination on Latinx persons, while acknowledging that discrimination is a product of structural racism shaping policies, institutional and organizational practices, and collective behavior. Our approach is strongly informed by theory. We drew from sociological theories of U.S. racial formation and Latinx racism (1, 15, 25, 26, 28, 36, 74), social epidemiology to acknowledge that racial and ethnic minorities embody inequality (181), legal studies such as intersectionality (178, 179), to acknowledge intersecting axes of oppression, and the biopsychosocial minority stress model (8, 9) to capture the acute and chronic stress and biobehavioral effects of racism. We assert the need to document and investigate the ways in which racism is harmful to Latinx persons' short-term and long-term health to develop institutional, organizational and individual interventions that mitigate the effects of racism on health, and ultimately, end racism.

We started this paper describing the complexities in the Latina/o/x population by nationality, migratory experience, generational status, phenotype, culture, and language, to emphasize that their experiences are diverse, and this variability should be captured in one's data. We believe that divergent findings about the relationship between racism and adverse health in Latinx populations (in comparison to more consistent findings among non-Hispanic Blacks) can be explained by the heterogeneities in Latinx persons' intersecting identities, how these are stratified in society, and their exposure to structural and institutional racism. Their biobehavioral responses to chronic racial stress will be diverse and how that manifests as chronic disease is vitally important given the growth of cardiometabolic health disparities in this population (e.g., prediabetes and T2DM) and the reality that racism globally is not diminishing. For example, biological sensitivity to context is different for recent Latinx immigrants to the U.S. in comparison to fourth generation Nuyoricans, in which the latter are more receptive to racial codes and may have adapted different coping strategies and physiological stress responses as they have been exposed to racist environments longer.

For this reason, we must know the history of diverse Latinx groups in the geographic areas in which we are conducting our research so that way we can contextualize sources of racism and trace them to larger systems, not just individuals' prejudiced behavior. The authors have conducted Latinx research in many U.S. cities and know that institutional racism takes different forms in different regions. For example, structural and institutional racism in the Southwestern United States is centered around the context of the U.S.-Mexico border. For this reason, we end by recommending that researchers contextualize the racial and ethnic identification and discrimination variables, with qualitative research or extant ethnic studies research. Describe discrimination measures, what race and ethnicity means for their sample, and how institutions (education, labor, immigration) under study produce racism that exacerbates the Latinx population's health. We need more research examining the health of the Latinx population in less populous states (e.g., new destinations) and non-urban areas, because most NIH-funded Latinx health research takes place in California, Texas, Northeastern U.S. cities, and Illinois (23).

While the present paper focused on specific biomarkers (i.e., allostatic stress processes) to exemplify the ways in which exposure to racism may impact the development of T2DM, there are other important biological processes that were not discussed yet warrant attention in future research conducted with Latinx communities. Specifically, biological indicators of human aging such as Leukocyte Telomere Length (LTL) may be another way in which racism gets under the skin and contributes to disease pathology (182). Recent research suggests exposure to discrimination is associated with shorter LTL among African American adults (183), suggesting discrimination experiences accelerate aging as measured by LTL, which may in turn contribute to disease burden in older adulthood.

There are challenges integrating biospecimens in embodiment of racism research because of historical and recent medical neglect and exploitation of Latinx groups. For example, the early clinical trials of oral contraceptives on Puerto Rican women (184) and the U.S. Public Health Service-funded Guatemalan Syphilis Study (185). Recently, women, mainly Latinx immigrants, detained in ICE detention centers were given long-term contraception, had their reproductive organs removed, and some were even sterilized without their consent (20). Moreover, the disparities in COVID-19 vaccinations, infections, deaths, and hospitalizations for Latinx persons across the United States demonstrates how structural racism in economics/labor, healthcare access, land/housing, politics, and civil rights work together to create the conditions for these inequities.

Biobehavioral research alone will not inform organizational and state policies as they sit in academic journals. It is important to develop this research to produce evidence and quality data that are not yet available for scholars to produce longitudinal research that demonstrates the effects of structural racism on Latinx persons' physical health. Moreover, researchers need to share the results of our research with local, state and federal policy makers so that they can know how their institutional practices and policies have biobehavioral consequences on Latinx subgroups. There is little to no data to capture the biobehavioral and long-term effects of state-sanctioned violence from immigration enforcement policies post-9/11 to the present, the implementation of the Affordable Care Act (186), segregation and displacement from economic development and gentrification (177), and police brutality.

We end by asserting that conducting more biobehavioral research is important because it provides powerful evidence of inequities that can be the catalyst for change. In the social sciences, there has been a hesitation to integrate biology, much less biospecimens into racism and health research because of the history of biological constructions of race that legitimized slavery, colonialism, and the medical exploitation of Black, Indigenous and other people of color. There are legitimate concerns that such research will further concretize racial categories as a biological reality. However, one of the most convincing forms of evidence that racism is harmful for population health has been biological evidence (5). Yet, the responsibility falls on researchers integrating and conducting biobehavioral research to use ethical and ecosocial approaches (7). More importantly, once the research is complete, we must communicate our findings to Latinx communities to verify their lived experiences and motivate them to civic engagement. We must also communicate the results from our biobehavioral racism to important stakeholders who can improve the institutional and structural conditions.

## Author Contributions

AM conceptualized the manuscript, engaged in investigation, wrote the original draft and figures, and reviewed and edited the manuscript. EM engaged in investigation, contributed to figures, and prepared the original draft. MB engaged in investigation, edited the figures, and reviewed and edited the manuscript. SK and DG validated our analysis and reviewed and edited the manuscript. All authors contributed to the article and approved the submitted version.

## Conflict of Interest

DG is the founder and chief scientific and strategy advisor at Salimetrics LLC and Salivabio LLC and that the nature of these relationships is managed by the policies of the committees on conflict of interest at the Johns Hopkins University School of Medicine and the University of California at Irvine. The remaining authors declare that the research was conducted in the absence of any commercial or financial relationships that could be construed as a potential conflict of interest.

## Publisher's Note

All claims expressed in this article are solely those of the authors and do not necessarily represent those of their affiliated organizations, or those of the publisher, the editors and the reviewers. Any product that may be evaluated in this article, or claim that may be made by its manufacturer, is not guaranteed or endorsed by the publisher.
